# ﻿Four new species of *Pichia* (Pichiales, Pichiaceae) isolated from China

**DOI:** 10.3897/mycokeys.114.142474

**Published:** 2025-02-26

**Authors:** Liang-Chen Guo, Shuang Hu, Hai-Yan Zhu, Yu-Jie Shang, Yan-Jie Qiu, Zhang Wen, Shen-Xi Chen, Feng-Yan Bai, Pei-Jie Han

**Affiliations:** 1 State Key Laboratory of Mycology, Institute of Microbiology, Chinese Academy of Sciences, Beijing 100101, China Institute of Microbiology, Chinese Academy of Sciences Beijing China; 2 College of Life Sciences, University of Chinese Academy of Sciences, Beijing 100049, China University of Chinese Academy of Sciences Beijing China; 3 School of Life Sciences, Institute of Life Sciences and Green Development, Hebei University, Baoding 071002, Hebei, China Hebei University Baoding China; 4 Hubei Key Laboratory of Quality and Safety of Traditional Chinese Medicine Health Food, Jing Brand Co. Ltd., Huangshi 435100, Hubei, China Hubei Key Laboratory of Quality and Safety of Traditional Chinese Medicine Health Food, Jing Brand Co. Ltd. Huangshi China

**Keywords:** New species, phylogeny, taxonomy, urease activity

## Abstract

The genus *Pichia* belonging to the family Pichiaceae is widely distributed worldwide and has garnered significant attention due to its importance in various industries and its potential role in human infections. During our investigation of yeast diversity in China, several strains representing undescribed taxa were isolated from forests in Hainan province, Hubei province, Beijing city and a mudflat in Guangdong province. Based on phylogenetic analyses of the internal transcribed spacer (ITS) region and the D1/D2 domain of the large subunit (LSU) rRNA, these strains were identified as four new species: *Pichiakregeriana***sp. nov.** (holotype strain CGMCC 2.7383^T^), *P.phaffii***sp. nov.** (holotype strain CGMCC 2.8239^T^), *P.ureolytica***sp. nov.** (holotype strain CGMCC 2.6825^T^) and *P.wuzhishanensis* f.a. **sp. nov.** (holotype strain CGMCC 2.7381^T^). The six strains of *P.ureolytica* were identified as positive for urease production. This phenomenon is extremely rare in the genus *Pichia*, as only *P.bovicola* is reported to exhibit weak urease activity.

## ﻿Introduction

In the past decade, approximately 400 research papers related to *Pichia* species have been published annually according to data from Web of Science (accessed on October 31, 2024). *Pichia* species have significant applications in various industries, including biosynthesis (Zhang et al. 2023), biotransformation (Ranieri et al. 2024), alcoholic and food fermentation (Teramoto et al. 2001; Hu et al. 2022; Xiao et al. 2023), feed production (Qvirist et al. 2016), enzyme production (Tsang 2011) and epidemiology (Yadav et al. 2012). Furthermore, besides the benefits mentioned above, the species *Candidakrusei*, a synonym of *Pichiakudriavzevii*, is one of the significant species, which can cause human infections even on the WHO fungal priority pathogens list (Nguyen et al. 2024).

The genus *Pichia*, which was previously classified under the order Saccharomycetales within the class Saccharomycetes, has now been transferred to the order Pichiales within the class Pichiomycetes by Groenewald et al. (2023). It was established by Hansen in 1904 with *Pichiamembranifaciens* as the type species. This genus is characterized by its multilateral budding, the occasional presence of pseudohyphae (not true hyphae), and the formation of ascospores that may be hat-shaped, hemispheroidal, or spherical, with or without a ledge (Kurtzman 2011). Almost all species within *Pichia* are capable of fermenting glucose, but do not assimilate nitrate (Kurtzman 2011). In the fourth edition of “*The Yeasts, a Taxonomic Study*,” Kurtzman (1998) accepted 91 species within the genus *Pichia*, which was traditionally considered polyphyletic. However, Kurtzman et al. (2008) later redefined the genus *Pichia* and clarified its phylogenetic relationships with genera such as *Issatchenkia* and *Williopsis*, using the large and small subunit (LSU and SSU) rRNA genes and the translation elongation factor-1a (*tef-1a*) gene. In the fifth edition of “*The Yeasts, a Taxonomic Study*,” Kurtzman (2011) recognized 20 species within the genus *Pichia*. Since then, several new species and new combinations have been reported in the genus *Pichia* (Bhadra et al. 2008; Limtong et al. 2009; Ganter et al. 2010; Sipiczki 2012; Ren et al. 2015; Kobayashi et al. 2017; Gao et al. 2018; Groenewald et al. 2018; Angchuan et al. 2022; Opulente et al. 2023; Chai et al. 2024; Zhu et al. 2024). For example, Zhu et al. (2024) described a novel species from marine sediment and transferred eight related *Candida* species to the genus *Pichia*. Chai et al. (2024) documented *Pichiateotihuacanensis* from a sample of the Mexican alcoholic beverage Pulque. The genus currently includes a total of 41 species (Suppl. material 1).

In this study, nine yeast strains were isolated from rotten wood in Hainan province, marine sediment in Guangzhou province, and bark samples in Hubei province and Beijing city. These strains were identified as four new species of the genus *Pichia* based on phylogenetic analysis and their morphological, physiological and biochemical characteristics, which contributed to enriching the diversity of *Pichia* in China.

## ﻿Materials and methods

### ﻿Sample collection and yeast isolation

A variety of samples for yeast isolation were collected from 2021 to 2023, including marine sediments from Dongguan city in Guangdong province, rotten wood from Wuzhishan city in Hainan province and bark from Shennongjia Forest Area in Hubei province and Mentougou district in Beijing city. Samples were sealed in 50 mL sterile centrifuge tubes and transported to the laboratory immediately for storage at 4 °C. Yeasts from rotten wood and bark samples were isolated using the enrichment method described by Yu et al. (2023). The isolation of yeast from marine sediments followed the methods described by Zhu et al. (2023). All strains were suspended in 25% glycerol and stored at –80 °C for long-term preservation.

### ﻿Morphological, physiological and biochemical studies

Morphological, physiological and biochemical properties were conducted according to standard methods established by Kurtzman et al. (2011). Carbon and nitrogen assimilation tests were performed in liquid media, with starved inoculum used for nitrogen testing. Sugar fermentation was tested in a liquid medium with Durham tubes. Growth at various temperatures (25, 30, 37 and 40 °C) was determined by cultivation on YPD agar. The formation of pseudohyphae was investigated using the Dalmau plate on corn meal agar (CMA: 2.5% corn starch and 2% agar) with sterile slides to create an anaerobic environment. The potential sexual cycles of strains were investigated using CMA, potato dextrose agar (PDA: 20% potato infusion, 2% glucose, and 2% agar), yeast carbon base agar (YCB: 1.17% yeast carbon base, 2% agar) and V8 agar (10% V8 juice and 2% agar). Each test strain was inoculated separately or mixed on agar plates and incubated at 25 °C for up to two months, which was observed once every half a month.

### ﻿DNA extraction, PCR amplification and sequencing

DNA of yeast cells was extracted using the method described by Wang and Bai (2008). The ITS region and D1/D2 domain were amplified with primers ITS1/ITS4 (White et al. 1990) and NL1/NL4 (Kurtzman and Robnett 1998), respectively. PCR products were commercially sequenced by Sangon Biotech Co., LTD. (Beijing, China) and the identity and accuracy of obtained nucleotide sequences were determined by comparing them to sequences in GenBank. All newly generated sequences were submitted to GenBank (https://www.ncbi.nlm.nih.gov/genbank/).

### ﻿Phylogenetic analyses

The sequences obtained in this study and reference sequences downloaded from GenBank (Suppl. material 1) were aligned using MAFFT v. 7 and manually improved using MEGA v. 7 where it was necessary (Katoh and Standley 2013; Kumar et al. 2016). Phylogenetic analysis based on single D1/D2 or ITS sequences was performed based on the evolutionary distance data calculated from Kimura’s two parameter model using the neighbour-joining algorithm in MEGA v. 7 (Kimura 1980; Kumar et al. 2016; Lachance 2022). Maximum-likelihood phylogenetic analysis based on the concatenated D1/D2 and ITS sequences was performed using the optional model GTR+I+G determined in MEGA v. 7 (Kumar et al. 2016). The confidence levels of the clades were estimated through 1000 replicates bootstrap analysis (Felsenstein 1985).

## ﻿Results

### ﻿Phylogenetic analyses

**Figure 1.**
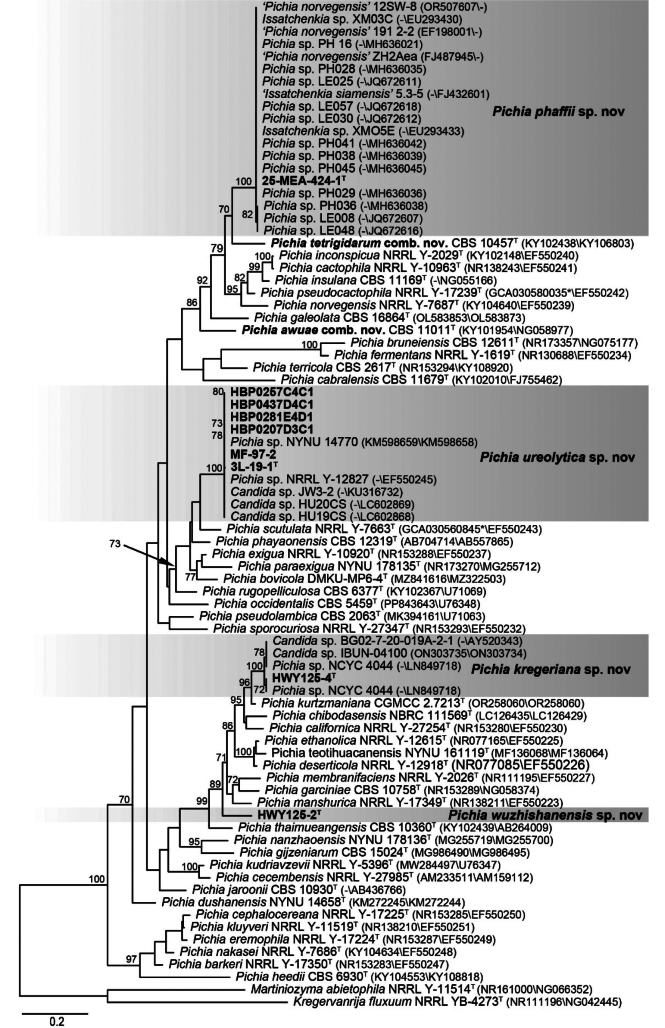
Maximum likelihood phylogenetic tree of the genus *Pichia* based on D1/D2 and ITS sequences. The species *Kregervanrijafluxuum* and *Martiniozymaabietophila* were used as the outgroup. Bootstrap values above 70% are shown on the branches. Type strains are denoted with the superscript ‘T’. Strains isolated in this study and the new combinations were marked in bold. Bar, 0.2 substitutions per nucleotide position.

Among the yeasts isolated from marine sediment, bark and rotten wood samples collected from different regions of China, nine yeast strains that could not be identified as any known species were selected for further taxonomic study. To determine the phylogenetic placement of the potential novel strains, phylogenetic analysis was conducted using the ITS and D1/D2 sequences of the nine strains and type strains of members in the genus *Pichia*. The phylogenetic trees indicated that these nine strains represented four new species in the *Pichia* clade (Fig. 1, Suppl. materials 2, 3).

Strain HWY125-4 from Hainan province formed a clade together with strains BG02-7-20-019A-2-1 from the USA, IBUN-04100 from Brazil, and NCYC 4038 and NCYC 4044 from Ecuador. These five strains exhibited one nucleotide difference in the D1/D2 domain. When the ITS sequence is available for comparison, strains HWY125-4 and IBUN-04100 possessed similar ITS sequences with no more than four nucleotide mismatches. The clade represented by type strain HWY125-4 is closely related to *P.kurtzmaniana* but differed from the type strain by 15 (2.8%, 11 substitutions and four gaps) and 35 (8.7%, 14 substitutions and 21 gaps) nucleotide mismatches in the D1/D2 domain and ITS region, respectively (Fig. 1, Suppl. materials 2, 3). The result suggested that the HWY125-4 clade represented a novel species in the genus *Pichia*.

Strain 25-MEA-424-1 from Guangdong province was grouped in a distinct clade along with 14 other unidentified strains and one previously identified as *Issatchenkiasiamensis* with only D1/D2 sequences available in GenBank (Suppl. material 2). The strains in this clade possess similar D1/D2 sequences with no more than one nucleotide difference (Suppl. material 2). Meanwhile, strain 25-MEA-424-1 formed a clade together with three other strains previously identified as *Pichianorvegensis* with only ITS sequences available in GenBank. These four strains in this clade possessed identical ITS sequences (Suppl. material 3). The strains in the 25-MEA-424-1 group previously named as *Issatchenkiasiamensis* and *Pichianorvegensis* (Suppl. material 1, Fig. 1) were obviously misidentifications. The name *Issatchenkiasiamensis* cannot be found in any global fungal name registration repositories, namely, Index Fungorum, Mycobank and Fungal Names, which is not recognized as a valid name. The group 25-MEA-424-1 differed from the closest species *Pichiatetrigidarum* comb. nov. by 31 (5.7%, 26 substitutions and five gaps) nucleotide mismatches and 61 (14.7%, 31 substitutions and 30 gaps) nucleotide mismatches in the D1/D2 domain and ITS region, respectively (Fig. 1, Suppl. materials 2, 3). These results suggested that the 25-MEA-424-1 clade represented a novel species in the genus *Pichia*.

Strains 3L-19-1 and MF-97-2 from Beijing city and HBP0207D3C1, HBP0257C4C1, HBP0281E4D1, and HBP0437D4C1 from Hubei province in this study as well as other five unidentified strains possessed similar D1/D2 sequences with no more than three nucleotide differences. Only the ITS sequence of NYNU14770 is available for comparison among the five strains. The ITS sequences of all these seven strains were similar with one nucleotide difference. The group represented by strain 3L-19-1 together with six other known species, *viz. P.bovicola*, *P.exigua*, *P.paraexigua*, *P.phayaonensis*, *P.rugopelliculosa*, and *P.scutulata* clustered in a branch with high support value. This group differed from the other species in this branch by 31~66 (5.8%~11.9%) nucleotide mismatches and 34~45 (9.4%~11.6%) nucleotide differences in the D1/D2 domain and ITS region, respectively. These results suggested that they represented a novel species in the genus *Pichia*.

Strain HWY125-2 from Hainan province formed a separate branch and did not cluster with any other strains (Fig. 1, Suppl. materials 2, 3). It differed from the closest species *Pichiathaimueangensis* by 35 (6.5%, 25 substitutions and 10 gaps) and 12 (5.4%, 7 substitutions and five gaps) nucleotide mismatches in the D1/D2 domain and ITS region, respectively (Fig. 1, Suppl. materials 2, 3). These results indicated that strain HWY125-2 represented a novel species in the genus *Pichia*.

### ﻿Taxonomy

#### ﻿ Pichia kregeriana

Taxon classificationFungiPichialesPichiaceae

S. Hu, L.C. Guo, F.Y. Bai & P.J. Han sp. nov.

BBC8A00D-AD3D-5B9A-88A6-4100F966A422

Fungal Names: FN 572224

##### Etymology.

The species *kregeriana* (kre.ge.ri.a’na. N.L. fem. adv.) is named in honor of the late Dr. Nelly Jeanne Wilhelmina Kreger-van Rij for her work in the field of microbiology, particularly in yeast systematics and ultrastructure.

##### Type.

The holotype CGMCC 2.7383 (original number = HWY125-4) was isolated from rotten wood of *Garciniamangostana* collected from Wuzhishan city, Hainan province, China (18.902°N, 109.688°E; tropical monsoon oceanic climate) by S. Hu in August, 2023 and had been deposited in a metabolically inactive state in the China General Microbiological Culture Collection Centre (CGMCC), Beijing, China. An ex-type culture had been deposited in the Japan Collection of Microorganisms (JCM), Koyadai, Japan, as JCM 36906. GenBank accessions: ITS-PQ586094 and LSU-PQ586304.

##### Culture characteristics.

After growth on YPD agar for 3 days at 25 °C, colonies are white, butyrous, nearly circular, lightly raised, rough and wrinkled with irregular surfaces and margins (Fig. 3A). Cells are oval-shaped or oval (3.3–4.5 × 4.9−8.7 μm) and budding is multilateral (Fig. 3B). No pseudohyphae are formed. Asci (2.4–4.6 × 5.0–5.4 um) are persistent and conjugated, typically forming two to four spherical spores within shuttle-shaped or triangular ascospores (Fig. 3C). The sexual structures were observed on YCB agar after 30 days at 25 °C. Conjugation can occur between a cell and its bud, or between independent cells.

##### Physiological and biochemical characteristics.

Glucose is not fermented. Glucose, ethanol, N-acetyl-D-glucosamine, glycerol (slow), D-glucosamine (weak), inulin (weak), sucrose (weak) and succinic acid are assimilated as sole carbon sources. D-galactose, L-sorbose, erythritol, D-xylose, glucitol, D-maltose, sodium citrate dihydrate, cellobiose, trehalose, lactose, melibiose, raffinose, melezitose, soluble starch, L-arabinose, D-arabinose, xylitol, D-ribose, L-rhamnose, methanol, ribitol, galactitol, D-mannitol, α-methyl-D-glucoside, salicin, D-glucuronic acid, DL-lactic acid, inositol and hexadecane are not assimilated as sole carbon sources. Ethylamine hydrochloride, cadaverine dihydrochloride, L-lysine and ammonium sulfate are assimilated as sole nitrogen sources. Potassium nitrate and sodium nitrite are not assimilated as sole nitrogen sources. Growth in vitamin-free medium and on 50% (w/v) glucose are positive, while growth in 10% NaCl plus 5% glucose medium and on 60% (w/v) glucose are negative. Diazonium blue B, urease activity and production of extracellular starch-like compounds are negative. Growth on YPD agar at 30 °C is positive, but negative at 37 °C.

##### Note.

*Pichiakregeriana* is physiologically differentiated from its closely related species, *Pichiakurtzmaniana*, by fermentation of glucose, growth on 60% (w/v) glucose and growth in 10% NaCl plus 5% glucose medium.

#### ﻿ Pichia phaffii

Taxon classificationFungiPichialesPichiaceae

H.Y. Zhu, L.C. Guo, F.Y. Bai & P.J. Han sp. nov.

C6AB4321-CD91-59DA-8D43-0345A904AC26

Fungal Names: FN 572225

##### Etymology.

This species *phaffii* (phaf’fi.i. N.L. gen. n.) is named in honor of the late Prof. Herman Phaff for his significant contributions to the field of microbiology, particularly in yeast systematics and ecology.

##### Type.

The holotype CGMCC 2.8239 (original number = 25-MEA-424-1) was isolated from mudflat soil sediment collected from Dongguan city, Guangdong province, China (23.042°N, 113.743°E; subtropical monsoon climate) by H.Y. Zhu in July 2022 and had been deposited in a metabolically inactive state in the China General Microbiological Culture Collection Centre (CGMCC), Beijing, China. GenBank accessions: ITS-PQ586092 and LSU-PQ586298.

##### Culture characteristics.

**Figure 2.**
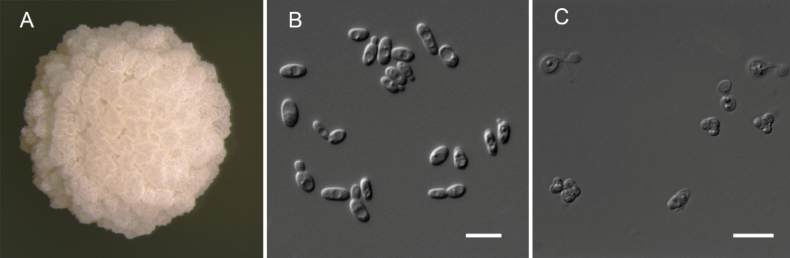
Morphology of *Pichiakregeriana* strain HWY125-4^T^. **A** Colony on YPD agar at 25 °C after 3 days **B** vegetative cells on YPD agar at 25 °C after 3 days **C** asci and ascospores on YCB agar at 25 °C after 30 days. Scale bars: 10 µm.

After growth on YPD agar for 3 days at 25 °C, colonies are white butyrous, rough and wrinkled with irregular surfaces and margins (Fig. 2A). Cells are ovoid to elongate (2.1–3.2 × 3.3–7.1 μm) and occur singly or in pairs (Fig. 2B). Budding is multilateral and pseudohyphae are formed (Fig. 2D). Asci (2.8–3.5 × 6.2–7.1 μm) are persistent and unconjugated, typically forming two to four spherical spores within diamond-shaped ascus ascospores, although a linear arrangement of ascospores is seen as well (Fig. 2C). The sexual structures were observed on YCB agar after 30 days at 25 °C.

**Figure 3.**
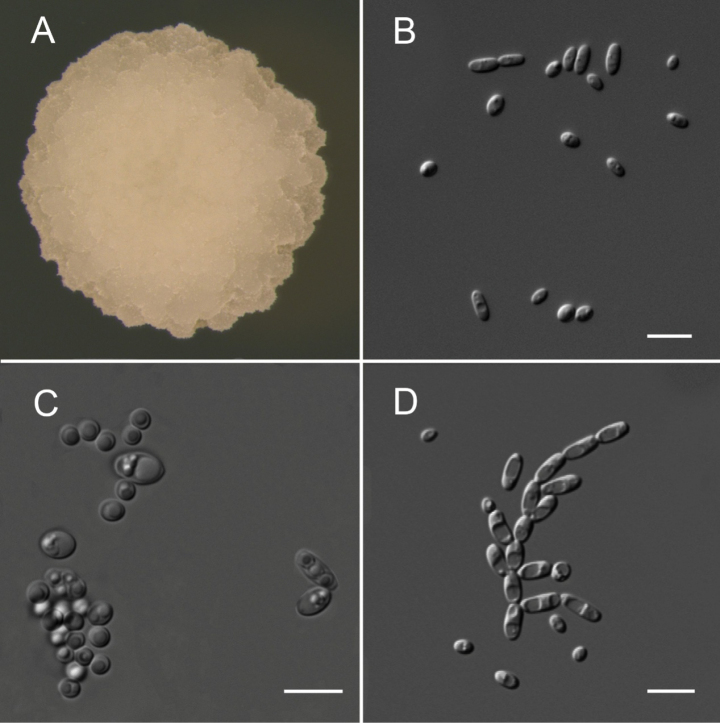
Morphology of *Pichiaphaffii* strain 25-MEA-424-1^T^. **A** Colony on YPD agar at 25 °C after 3 days **B** vegetative cells on YPD agar at 25 °C after 3 days **C** asci and ascospores on YCB agar at 25 °C after 30 days **D** pseudohyphae on YPD agar at 25 °C after 15 days. Scale bars: 10 µm.

##### Physiological and biochemical characteristics.

Glucose is not fermented. Glucose, ethanol, erythritol (weak), succinic acid, sodium citrate dihydrate (weak), D-glucosamine, inulin (weak) and DL-lactic acid are assimilated as sole carbon sources. D-galactose, L-sorbose, D-xylose, glycerol, glucitol, sucrose, maltose, cellobiose, trehalose, lactose, melibiose, raffinose, melezitose, soluble starch, N-acetyl-D-glucosamine, L-arabinose, D-arabinose, xylitol, D-ribose, L-rhamnose, methanol, ribitol, galactitol, D-mannitol, α-methyl-D-glucoside, salicin, D-glucuronic acid, citrate acid, inositol and hexadecane are not assimilated as sole carbon sources. Ethylamine hydrochloride, cadaverine dihydrochloride, L-lysine, and ammonium sulfate are assimilated as sole nitrogen sources. Potassium nitrate and sodium nitrite are not assimilated as sole nitrogen sources. Growth in vitamin-free medium and on 50% (w/v) glucose are positive, while growth in 10% NaCl plus 5% glucose medium and on 60% (w/v) glucose is negative. Diazonium blue B, urease activity and production of extracellular starch-like compounds are negative. Growth on YPD agar at 30 °C is positive, but negative at 37 °C.

##### Notes.

*Pichiaphaffii* sp. nov. is physiologically differentiated from its closely related species *Pichiatetrigidarum* comb. nov. in that *Pichiatetrigidarum* comb. nov. can ferment D-glucose, D-galactose, sucrose, maltose, trehalose, while *Pichiaphaffii* sp. nov. does not.

#### ﻿ Pichia ureolytica

Taxon classificationFungiPichialesPichiaceae

L.C. Guo, Y.J. Shang, F.Y. Bai & P.J. Han sp. nov.

E7AEBDD7-D961-5210-A6F9-CF408410D72C

Fungal Names: FN 572226

##### Etymology.

The specific epithet *ureolytica* (u.re.o.ly’ti.ca. L. fem. adj.) derived from “urea” (the compound that can be broken down) and “lyticus,” which indicates the ability to break down or decompose urea.

##### Type.

The holotype CGMCC 2.6825 (original number = 3L-19-1) was isolated from bark of *Quercuswutaishansea* collected from Mentougou district, Beijing city, China (39.866°N; 115.598°E; warm temperate semi-humid and semi-arid monsoon climate) by Y.J. Shang in August 2021 and had been deposited in a metabolically inactive state in the China General Microbiological Culture Collection Centre (CGMCC), Beijing, China. The ex-type culture had been deposited in the Japan Collection of Microorganisms (JCM), Koyadai, Japan, as JCM 36370. GenBank accessions: ITS-PQ586091 and LSU-PQ586297.

##### Culture characteristics.

**Figure 4.**
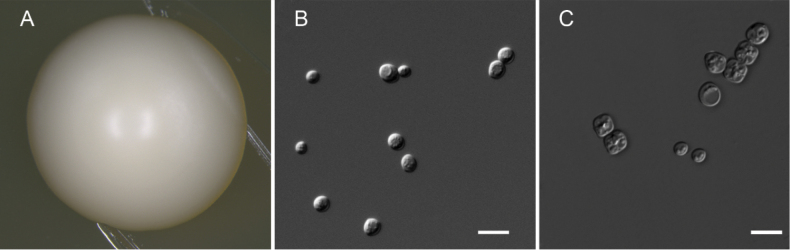
Morphology of *Pichiaureolytica* strain 3L-19-1^T^. **A** Colony on YPD agar at 25 °C after 3 days **B** vegetative cells on YPD agar at 25 °C after 3 days **C** asci and ascospores on YCB agar at 25 °C after 30 days. Scale bars: 10 µm.

After growth on YPD agar for 3 days at 25 °C, colonies are white, circular, butyrous and smooth with entire margins (Fig. 4A). Cells are ovoid (3.5–5.0 × 3.8–5.2 μm) and occur singly or in pairs. Budding is multilateral and pseudohyphae are not formed (Fig. 4B). Asci (4.7–5.4 × 5.5–6.2 μm) are persistent, typically forming four spherical spores within a diamond-shaped or subrounded ascus. (Fig. 4C). The sexual structures were observed on YCB agar after 30 days at 25 °C.

##### Physiological and biochemical characteristics.

Glucose fermentation is weak. Glucose, ethanol, succinic acid (slow/weak) and DL-lactic acid are assimilated as sole carbon sources. Galactose, L-sorbose, D-xylose, D-glucosamine, glycerol, glucitol, sucrose, maltose, cellobiose, trehalose, lactose, melibiose, raffinose, melezitose, inulin, soluble starch, N-acetyl-D-glucosamine, L-arabinose, D-arabinose, xylitol, D-ribose, L-rhamnose, methanol, erythritol, ribitol, galactitol, D-mannitol, α-methyl-D-glucoside, salicin, D-glucuronic acid, sodium citrate dihydrate, inositol and hexadecane are not assimilated as sole carbon sources. Ethylamine hydrochloride, cadaverine dihydrochloride, L-lysine and ammonium sulfate are assimilated as sole nitrogen sources. Potassium nitrate and sodium nitrite are not assimilated as sole nitrogen sources. Growth in vitaminfree medium and on 50% and 60% (w/v) glucose are positive, while growth in 10% NaCl plus 5% glucose medium is negative. Urease activity is positive (Suppl. material 4). Diazonium blue B reaction and production of extracellular starchlike compounds are negative. Growth on YPD agar at 30 °C is positive, but negative at 37 °C.

##### Notes.

*Pichiaureolytica* is physiologically differentiated from its closely related species *Pichiaparaexigua* and *Pichiascutulata* in terms of urease activity; from its closely related species *Pichiabovicola* and *Pichiaphayaonensis* by growth on 60% (w/v) glucose; from its closely related species *Pichiaexigua* and *Pichiaoccidentalis* by the fact that *Pichiaexigua* and *Pichiaoccidentalis* are capable of growth at 37 °C, whereas *Pichiaureolytica* does not show growth under the same temperature conditions.

#### ﻿ Pichia wuzhishanensis

Taxon classificationFungiPichialesPichiaceae

f.a. S. Hu, L.C. Guo, F.Y. Bai & P.J. Han sp. nov.

719BC3C1-3C6C-5A73-BCFB-234CA5C4A2ED

Fungal Names: FN 572227

##### Etymology.

The specific epithet *wuzhishanensis* (wu.zhi.han’en.sis. N.L. fem. adj.) is named after the location where the type strain of the species was isolated: Wuzhishan city, Hainan province, China.

##### Type.

The holotype CGMCC 2.7381 (original number = HWY125-2) was isolated from rotten wood of *Garciniamangostana* collected from Wuzhishan city, Hainan province, China (18.902°N, 109.688°E; tropical monsoon oceanic climate) by S. Hu in August, 2023 and had been deposited in a metabolically inactive state in the China General Microbiological Culture Collection Centre (CGMCC), Beijing, China. An ex-type culture had been deposited in the Japan Collection of Microorganisms (JCM), Koyadai, Japan, as JCM 36905. GenBank accessions: ITS-PQ586093 and LSU-PQ586303.

##### Culture characteristics.

**Figure 5.**
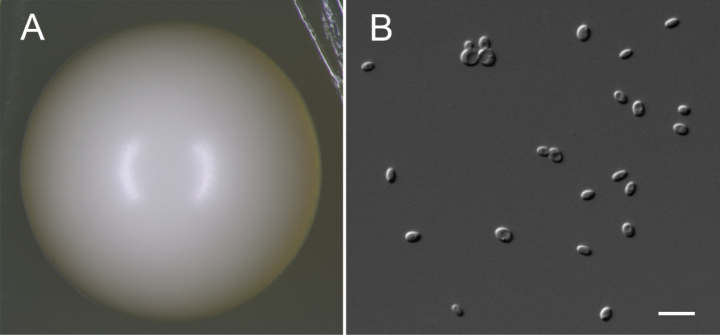
Morphology of *Pichiawuzhishanensis* strain HWY125-2^T^. **A** Colony on YPD agar at 25 °C after 3 days **B** vegetative cells on YPD agar at 25 °C after 3 days. Scale bars: 10 µm.

After growth on YPD agar for 3 days at 25 °C, colonies are white, butyrous, circular and glossy, with a smooth surface and entire margins (Fig. 5A). Cells are oval-shaped or oval (2.1–4.5 × 3.1–5.7 μm) and budding is monopolar (Fig. 5B). Sexual structures were not observed in single or mixed strain cultures on CMA, PDA and V8 agar after 2 months of incubation at 25 °C.

##### Physiological and biochemical characteristics.

Glucose is not fermented. Glucose, ethanol, N-acetyl-D-glucosamine, glycerol, D-glucosamine, succinic acid and DL-lactic acid (weak) are assimilated as sole carbon sources. D-galactose, L-sorbose, erythritol, D-xylose, glucitol, maltose, sodium citrate dihydrate, cellobiose, trehalose, lactose, melibiose, raffinose, melezitose, soluble starch, L-arabinose, D-arabinose, xylitol, D-ribose, L-rhamnose, methanol, ribitol, galactitol, D-mannitol, α-methyl-D-glucoside, salicin, D-glucuronic acid, inositol, sucrose, inulin and hexadecane are not assimilated as sole carbon sources. Ethylamine hydrochloride, cadaverine dihydrochloride, L-lysine and ammonium sulfate are assimilated as sole nitrogen sources. Sodium nitrite and potassium nitrate are not assimilated as sole nitrogen sources. Growth in vitamin-free medium and on 50% (w/v) glucose are positive, while growth in 10% NaCl plus 5% glucose medium and on 60% (w/v) glucose are negative. Diazonium blue B, urease activity and production of extracellular starch-like compounds are negative. Growth on YPD agar at 37 °C is positive, but negative at 42 °C.

##### Notes.

*Pichiawuzhishanensis* is physiologically differentiated from its closely related species *Pichiathaimueangensis* by its ability to assimilate D-xylose.

### ﻿New combinations

Although the sexual state of the new species, represented by the single strain HWY125-2, was not observed on any agar medium, following the Shenzhen Code (Turland et al. 2018), we classify the new species within the genus *Pichia* and propose the name *Pichiakregeriana* sp. nov. for it. Similarly, we reassign *Candidaawuae* and *Candidatetrigidarum*, which are robustly supported as members of the *Pichia* clade in the phylogenetic analyses (Fig. 1, Suppl. materials 2, 3) to the genus *Pichia*.

#### ﻿ Pichia awuae

Taxon classificationFungiPichialesPichiaceae

(D.S. Nielsen, M. Jakobsen & L. Jespersen) S. Hu, L.C. Guo, F.Y. Bai & P.J. Han comb. nov.

6C618B45-3C2D-5BAE-9991-05988EAA5FDB

Fungal Names No: FN 572228

##### Basionym.

*Candidaawuae* D.S. Nielsen, M. Jakobsen & L. Jespersen, International Journal of Systematic and Evolutionary Microbiology 60, 1460 (2010).

#### ﻿ Pichia tetrigidarum

Taxon classificationFungiPichialesPichiaceae

(S.O. Suh, N.H. Nguyen & M. Blackw) S. Hu, L.C. Guo, F.Y. Bai & P.J. Han comb. nov.

D9C8309A-666F-56B4-845F-364669C0CD41

Fungal Names No: FN 572229

##### Basionym.

*Candidatetrigidarum* S.O. Suh, N.H. Nguyen & M. Blackw., FEMS Yeast Research 8(1): 97 (2008).

## ﻿Discussions

In the present study, nine yeast strains from eight samples were isolated from various sources in different regions of China during the collection trips for yeast diversity conducted in 2021−2023. Four novel *Pichia* species, *P.kregeriana* sp. nov., *P.phaffii* sp. nov., *P.ureolytica* sp. nov., and *P.wuzhishanensis* f.a. sp. nov., were described from these strains based on a single-segment (D1/D2 or ITS) and a two-segment (ITS and D1/D2) approach, morphological, physiological and biochemical characteristics comparison. Notably, the two new species *P.wuzhishanensis* sp. nov. and *P.kregeriana* sp. nov. were isolated from the same rotten wood sample, which suggests that there may be many unknown microorganisms including novel yeast species in the environment around us waiting to be discovered and studied.

Urea is the main nitrogen-containing product of protein metabolism and decomposition in mammals, amphibians, and certain fish. Urea is not only present in urine, but also in serum, sweat, and exocrine gland secretions in humans. It can be used as a fertilizer, animal feed, explosive, glue stabilizer, and chemical raw material. And urease increases the decomposition rate of urea by 10^14^ times, promoting nitrogen assimilation back to amino acids, thus playing a crucial role in the global nitrogen cycle (Loharch and Berlicki 2022). In addition, urease is a virulence factor found in many pathogenic bacteria (Cox et al. 2000; Subramaniyan et al. 2023). Basidiomycetous yeasts with few exceptions are capable of hydrolyzing urea but most of them are unable to ferment glucose, whereas ascomycetous yeasts exhibit the opposite characteristics (Moore 1980). The ascomycetous yeasts *Schizosaccharomycespombe* and *Lipomyces* species are exceptions with positive urease activity (Kurtzman et al. 2011). In this study, the six strains isolated from China of the new species *Pichiaureolytica* sp. nov. were identified as positive for production of urease (Suppl. material 4). This phenomenon is extremely rare in the genus *Pichia*, and only *Pichiabovicola* is reported to indicate weak urease activity (Suppl. material 1).

Species of *Pichia* are ubiquitous and exist in various environments around the world (Suppl. material 1). In this study, the 19 strains representing *Pichiaphaffii* sp. nov. are from various sources across different countries, such as digestive tract of insect in Brazil, mangrove and marine sediment in China, and artificial lake sediment or water in Colombia; the 11 strains representing *Pichiaureolytica* sp. nov. originate from diverse locations, including bark in China, soil in Korea, and artificial lake sediment or water in Colombia; and the 5 strains representing *Pichiakregeriana* sp. nov. are sourced from rotten wood in China and gut of insect in USA. *Pichiakurtzmaniana*, as described by Zhu et al (2024), is also found in various environments, such as soil and marine water in China, soil in Brazil, deteriorated strawberry soft drinks in the UK, and fermentation broth and black viscous substances in Japan. Apart from these mentioned countries, species of *Pichia* are also found in Mexico (Chai et al. 2024); Thailand (Limtong et al. 2009), Indonesia (Kobayashi et al. 2017), the Netherlands (Groenewald et al. 2018), India (Bhadra et al. 2007), Caribbean (Ganter et al. 2010), Indonesia (Kobayashi et al. 2017), Japan (Ninomiya et al. 2010), Spain (Flórez et al. 2010), Borneo (Sipiczki 2012). In addition to natural environments, novel *Pichia* species have been identified in fermentation environments, including alcoholic beverages and blue-veined Cabrales cheese.

## Supplementary Material

XML Treatment for Pichia kregeriana

XML Treatment for Pichia phaffii

XML Treatment for Pichia ureolytica

XML Treatment for Pichia wuzhishanensis

XML Treatment for Pichia awuae

XML Treatment for Pichia tetrigidarum

## ﻿Additional information

### Author ORCIDs

Liang-Chen Guo https://orcid.org/0009-0004-9404-4449

Shuang Hu https://orcid.org/0000-0001-7285-7626

Hai-Yan Zhu https://orcid.org/0000-0001-5547-4922

Yu-Jie Shang https://orcid.org/0009-0000-2321-0332

Yan-Jie Qiu https://orcid.org/0009-0008-8397-9766

Feng-Yan Bai https://orcid.org/0000-0001-9823-6430

Pei-Jie Han https://orcid.org/0000-0001-8342-739X

### Data availability

The new sequences generated in this study have been deposited in GenBank (https://www.ncbi.nlm.nih.gov/genbank/) under accession numbers indicated in Suppl. material 1 and released to the public. All new taxa have been registered in Fungal Names (https://nmdc.cn/fungalnames/). The type strains of the new species described have been deposited in the China General Microbiological Culture Collection Center (CGMCC) with strain numbers indicated in Suppl. material 1.

## Supplementary materials

Supplementary material 1 The yeast species and strains were employed in this study Data type xlsx https://binary.pensoft.net/file/1263227This dataset is made available under the Open Database License (http://opendatacommons.org/licenses/odbl/1.0/). The Open Database License (ODbL) is a license agreement intended to allow users to freely share, modify, and use this Dataset while maintaining this same freedom for others, provided that the original source and author(s) are credited.Liang-Chen Guo, Shuang Hu, Hai-Yan Zhu, Yu-Jie Shang, Yan-Jie Qiu, Zhang Wen, Shen-Xi Chen, Feng-Yan Bai, Pei-Jie Han

Supplementary material 2 Neighbor-Joining phylogenetic tree of the genus *Pichia* based on the D1/D2 sequences Data type tif Explanation note Bootstrap values above 70% are shown on the branches. The species *Kregervanrijafluxuum* and *Martiniozymaabietophila* were used as the outgroup. Type strains are denoted with the superscript ‘T’. Strains isolated in this study and the new combinations were marked in bold. Bars, 0.02 substitutions per nucleotide position. https://binary.pensoft.net/file/1263228This dataset is made available under the Open Database License (http://opendatacommons.org/licenses/odbl/1.0/). The Open Database License (ODbL) is a license agreement intended to allow users to freely share, modify, and use this Dataset while maintaining this same freedom for others, provided that the original source and author(s) are credited.Liang-Chen Guo, Shuang Hu, Hai-Yan Zhu, Yu-Jie Shang, Yan-Jie Qiu, Zhang Wen, Shen-Xi Chen, Feng-Yan Bai, Pei-Jie Han

Supplementary material 3 Neighbor-Joining phylogenetic tree of the genus *Pichia* based on the ITS sequences Data type tif Explanation note Bootstrap values above 70% are shown on the branches. The species *Kregervanrijafluxuum* and *Martiniozymaabietophila* were used as the outgroup. Type strains are denoted with the superscript ‘T’. Strains isolated in this study and the new combinations were marked in bold. Bars, 0.02 substitutions per nucleotide position. https://binary.pensoft.net/file/1263229This dataset is made available under the Open Database License (http://opendatacommons.org/licenses/odbl/1.0/). The Open Database License (ODbL) is a license agreement intended to allow users to freely share, modify, and use this Dataset while maintaining this same freedom for others, provided that the original source and author(s) are credited.Liang-Chen Guo, Shuang Hu, Hai-Yan Zhu, Yu-Jie Shang, Yan-Jie Qiu, Zhang Wen, Shen-Xi Chen, Feng-Yan Bai, Pei-Jie Han

Supplementary material 4 Christensen agar plate for three days Data type tif Explanation note (a) Negative for *Saturnisporasinensis* strain CGMCC 2.6937. (b) Positive for *Vishniacozymapseudocarnescens* strain CGMCC 2.6457. (c) Positive for *Vishniacozymapseudodimennae* strain CGMCC 2.6790. (d) Positive for *Pichiaureolytica* 3L-19-1. (e) Positive for *Pichiaureolytica* MF-97-2. (f) Positive for *Pichiaureolytica* HBP0207D3C1. (g) Positive for *Pichiaureolytica* HBP0437D4C1. (h) Positive for *Pichiaureolytica* HBP0257C4C1. (i) Positive for *Pichiaureolytica* HBP0281E4D1. https://binary.pensoft.net/file/1263230This dataset is made available under the Open Database License (http://opendatacommons.org/licenses/odbl/1.0/). The Open Database License (ODbL) is a license agreement intended to allow users to freely share, modify, and use this Dataset while maintaining this same freedom for others, provided that the original source and author(s) are credited.Liang-Chen Guo, Shuang Hu, Hai-Yan Zhu, Yu-Jie Shang, Yan-Jie Qiu, Zhang Wen, Shen-Xi Chen, Feng-Yan Bai, Pei-Jie Han
