# Supplementary figures and images for: ﻿Four new species of Pichia (Pichiales, Pichiaceae) isolated from China
Source: MycoKeys. 2025 Feb 26;114:115–32. doi: 10.3897/mycokeys.114.142474 (PMC11883644; doi:10.3897/mycokeys.114.142474)

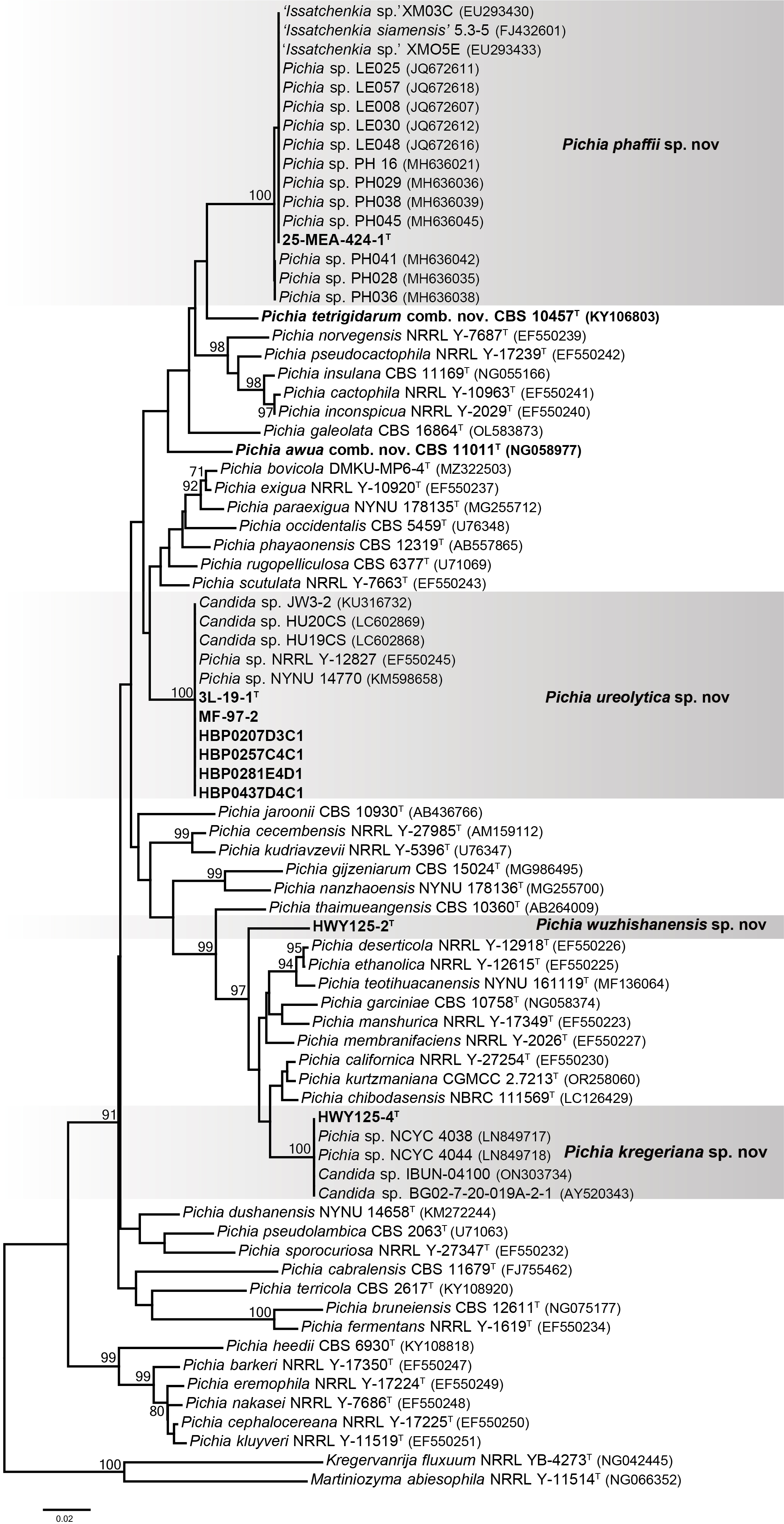

Supplement: Supplementary material 2 — Neighbor-Joining phylogenetic tree of the genus Pichia based on the D1/D2 sequences [file mycokeys-114-115-s002.tif]

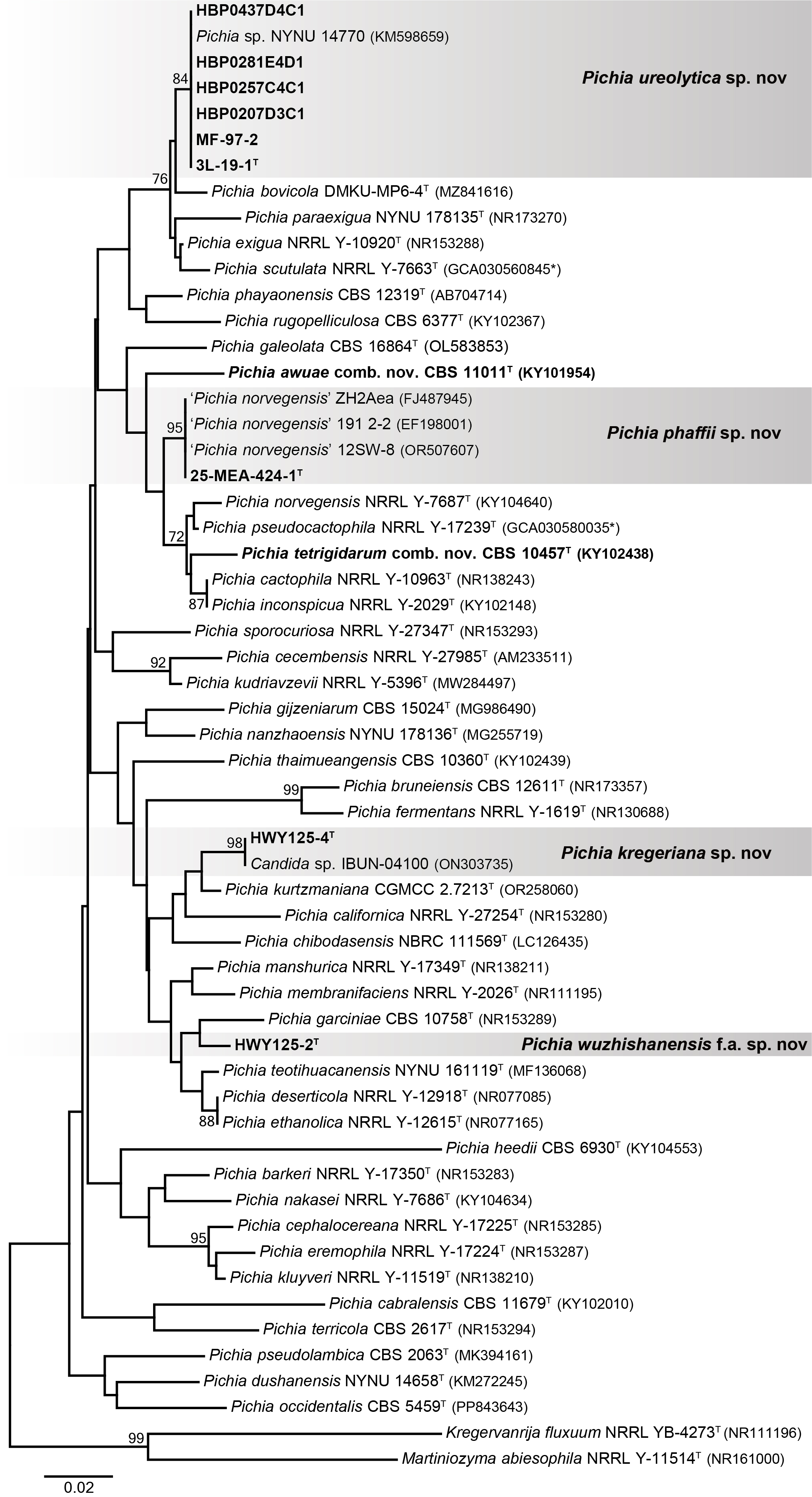

Supplement: Supplementary material 3 — Neighbor-Joining phylogenetic tree of the genus Pichia based on the ITS sequences [file mycokeys-114-115-s003.tif]

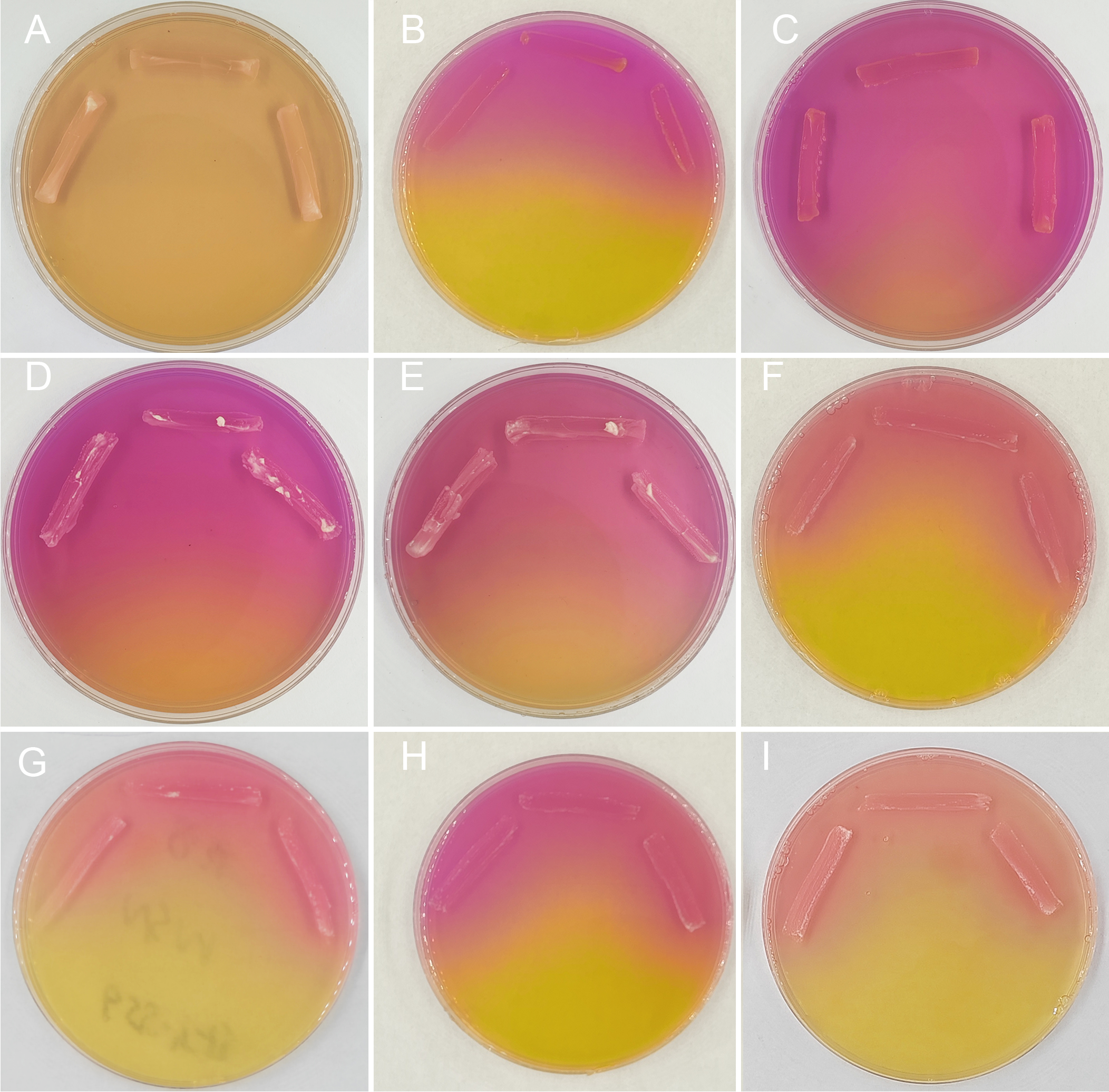

Supplement: Supplementary material 4 — Christensen agar plate for three days [file mycokeys-114-115-s004.tif]
